# ICTV Virus Taxonomy Profile: *Partitiviridae*

**DOI:** 10.1099/jgv.0.000985

**Published:** 2017-12-07

**Authors:** Eeva J. Vainio, Sotaro Chiba, Said A. Ghabrial, Edgar Maiss, Marilyn Roossinck, Sead Sabanadzovic, Nobuhiro Suzuki, Jiatao Xie, Max Nibert, ICTV Report Consortium

**Affiliations:** ^1^​ Natural Resources Institute Finland (Luke), Helsinki 00790, Finland; ^2^​ Asian Satellite Campuses Institute, Nagoya University, Nagoya 464-0861, Japan; ^3^​ Department of Plant Pathology, University of Kentucky, Lexington, KY 40546, USA; ^4^​ Institute of Horticultural Production Systems, Leibniz University Hannover, Hannover 30419, Germany; ^5^​ Center for Infectious Disease Dynamics, Pennsylvania State University, University Park, PA 16802, USA; ^6^​ Department of Biochemistry, Molecular Biology, Entomology and Plant Pathology, Mississippi State University, MS 39762, USA; ^7^​ Institute of Plant Science and Resources, Okayama University, Chuo 2-20-1, Kurashiki, 710-0046, Japan; ^8^​ College of Plant Science and Technology, Huazhong Agricultural University, Wuhan 430070, Hubei Province, PR China; ^9^​ Department of Microbiology and Immunobiology, Harvard Medical School, Boston, MA 02115, USA

**Keywords:** *Partitiviridae*, ICTV, taxonomy, *Alphapartitivirus*, *Betapartitivirus*, *Deltapartitivirus*, *Gammapartitivirus*, *Cryspovirus*

## Abstract

The *Partitiviridae* is a family of small, isometric, non-enveloped viruses with bisegmented double-stranded (ds) RNA genomes of 3–4.8 kbp. The two genome segments are individually encapsidated. The family has five genera, with characteristic hosts for members of each genus: either plants or fungi for genera *Alphapartitivirus* and *Betapartitivirus*, fungi for genus *Gammapartitivirus*, plants for genus *Deltapartitivirus* and protozoa for genus *Cryspovirus*. Partitiviruses are transmitted intracellularly via seeds (plants), oocysts (protozoa) or hyphal anastomosis, cell division and sporogenesis (fungi); there are no known natural vectors. This is a summary of the International Committee on Taxonomy of Viruses (ICTV) Report on the taxonomy of the *Partitiviridae*, which is available at www.ictv.global/report/partitiviridae.

## Virion

**Fig. 1.**
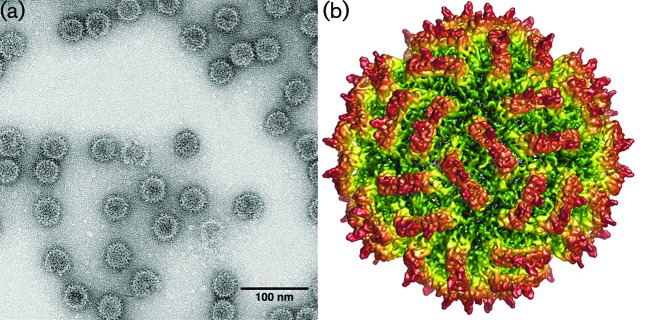
(a) Transmission electron micrograph of negatively-stained purified particles of Penicillium stoloniferum virus S, a representative member of the genus *Gammapartitivirus*. (b) Cryo-EM reconstructions of Penicillium stoloniferum virus S at 0.45 nm resolution, and rendered with radial colour mapping.

Virus particles are isometric, non-enveloped, and 25–43 nm in diameter (Table 1, Fig. 1a, b). Each capsid is composed of 120 copies of a single protein arranged as 60 dimers with *T*=1 icosahedral symmetry [1]. Dimeric surface protrusions are frequently observed on viral capsids. One or two molecules of RNA-dependent RNA polymerase (RdRP) are packaged inside each particle [2].

**Table 1.** Characteristics of the family *Partitiviridae*

**Typical member:**	Atkinsonella hypoxylon virus, 2H (RNA1, L39125; RNA2, L39126), species *Atkinsonella hypoxylon virus*, genus *Betapartitivirus*
Genome	3–4.8 kbp of linear bisegmented dsRNA
Virion	Isometric, non-enveloped, 25–43 nm in diameter; dsRNA1 and dsRNA2 are separately encapsidated
Replication	Cytoplasmic. Genomic RNA acts as a template for mRNA synthesis within the virus particle; transcription occurs by a semiconservative mechanism
Translation	From monocistronic positive-sense transcripts of both genomic dsRNAs
Host range	Plants, fungi and protozoa
Taxonomy	Five genera, including >40 species, and 15 species unassigned to a genus

## Replication

Each dsRNA is monocistronic. The RdRP is believed to function as both a transcriptase and a replicase and catalyzes *in vitro* end-to-end transcription of each dsRNA to produce mRNA by a semi-conservative mechanism. Virions accumulate in the cytoplasm.

## Genome

**Fig. 2.**
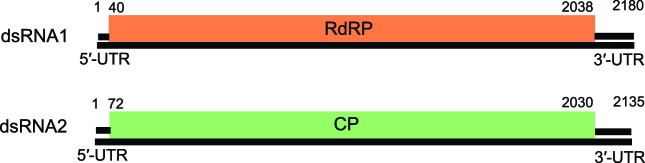
Atkinsonella hypoxylon virus [10], an isolate of the type species of the genus *Betapartitivirus*, has a bipartite genome consisting of dsRNA1 and dsRNA2.

Members of all five genera possess two essential genome segments, dsRNA1 and dsRNA2, each containing one large ORF on the positive-strand RNA molecule (Fig. 2). The smaller of the two dsRNA genome segments usually encodes the coat protein (CP) and the larger usually encodes the virion-associated RNA polymerase. The linear dsRNA segments are separately encapsidated. Additional dsRNA segments (satellite or defective) may also be present.

## Taxonomy

### Alphapartitivirus

Members of the genus *Alphapartitivirus* infect either plants, or ascomycetous or basidiomycetous fungi. The two essential dsRNA genome segments are individually about 1.9–2.0 kbp (dsRNA1) and 1.7–1.9 kbp (dsRNA2), typically containing a poly(A) tract near the plus-strand 3′-terminus. There is a single major CP with predicted *Mr* of 51–57 kDa. Plant alphapartitiviruses cause persistent infections, whereas some fungal alphapartitiviruses cause host effects, such as hypovirulence or a reduced growth rate [3, 4].

### Betapartitivirus

Members of the genus *Betapartitivirus* infect either plants, or ascomycetous or basidiomycetous fungi. The two essential dsRNA genome segments are about 2.2–2.4 kbp (dsRNA1) and 2.1–2.4 kbp (dsRNA2), typically containing a poly(A) tract near the plus-strand 3′-terminus. There is a single major CP with predicted *Mr* of 71–77 kDa. Plant betapartitiviruses cause persistent infections [5, 6]. Some fungal betapartitiviruses cause reduced host virulence and changes in colony morphology [7].

### Gammapartitivirus

All known members of the genus *Gammapartitivirus* infect ascomycetous fungi. The two essential dsRNA segments are about 1.6–1.8 kbp (dsRNA1) and 1.4–1.6 kbp (dsRNA2). There is a single major CP with predicted *Mr* of 44–47 kDa. Most gammapartitiviruses seem to induce latent infections. Aspergillus fumigatus partitivirus 1, a related, unclassified virus, has been associated with host effects.

### Deltapartitivirus

All known members of the genus *Deltapartitivirus* induce persistent infections in plants [8]. They are transmitted by ovule and pollen to the seed embryo. The two essential dsRNA segments are individually 1.6–1.7 kbp (dsRNA1) and 1.4–1.6 kbp (dsRNA2). There is a single major CP with predicted *Mr* of 38–49 kDa.

### Cryspovirus

Members of the genus *Cryspovirus* infect apicomplexan protozoa of the genus *Cryptosporidium* [9]. The viral genome comprises two dsRNA segments, which are individually 1.5 and 1.8 kbp. There is a single major CP with predicted *Mr* of 37 kDa. Virions are disseminated within *Cryptosporidium* oocysts. Infections of the *Cryptosporidium* host cells appear to be latent.

## Resources

Full ICTV Online (10th) Report: www.ictv.global/report/partitiviridae.
